# Efficacy and safety of golimumab 52-week maintenance therapy in Japanese patients with moderate to severely active ulcerative colitis: a phase 3, double-blind, randomized, placebo-controlled study-(PURSUIT-J study)

**DOI:** 10.1007/s00535-017-1326-1

**Published:** 2017-03-21

**Authors:** Toshifumi Hibi, Yuya Imai, Asako Senoo, Kentaro Ohta, Yoshifumi Ukyo

**Affiliations:** 1 Center for Advanced IBD Research and Treatment, Kitasato Institute Hospital, Kitasato University, 5-9-1 Shirokane, Minato-ku, Tokyo, 108-8462 Japan; 2 Janssen Pharmaceutical K.K., Tokyo, Japan

**Keywords:** Anti-TNFα antibody, Golimumab, Japanese patients, PURSUIT, Ulcerative colitis, Maintenance therapy

## Abstract

**Background:**

The global phase 3 studies of golimumab [PURSUIT-SC and PURSUIT-maintenance (M)], an anti-tumor necrosis factor-α (anti-TNFα) antibody, have demonstrated clinical efficacy and safety as induction and maintenance therapies in patients with moderate to severely active ulcerative colitis (UC). This study aimed to evaluate the efficacy and safety of golimumab as maintenance therapy in the Japanese population.

**Methods:**

In this phase 3, double-blind (DB), placebo-controlled, parallel group, randomized withdrawal study, 144 Japanese patients with moderately to severely active UC received golimumab doses of 200 mg (at week 0) and 100 mg (at week 2) subcutaneously during the 6-week open-label induction phase. Patients who responded to golimumab induction therapy entered the DB maintenance (M) phase and were randomized (1:1) to receive 100 mg of golimumab subcutaneous injection (SC) or placebo every 4 weeks for 52 weeks. The primary endpoint was clinical response through M-week 54; secondary endpoints included clinical remission and mucosal healing at M-week 30 and 54.

**Results:**

Among induction responders, more patients on golimumab treatment (56.3%) maintained clinical response through M-week 54 versus the placebo group (19.4%). At both M-week 30 and 54, 50% golimumab-treated patients achieved clinical remission versus the placebo group (6.5%) and a higher proportion of patients on golimumab (59.4%) experienced mucosal healing than the placebo group (16.1%). Incidence of treatment-emergent adverse events was 96.9% in the golimumab group and 71% in the placebo group. Overall, the efficacy and safety results in this study were comparable with those observed in global studies.

**Conclusions:**

Golimumab SC treatment maintained clinical efficacy through week 54 among induction responders, and no new safety signals were observed in the patients with moderate to severely active UC.

Clinical Trial Registration: The study is registered at ClinicalTrials.gov NCT01863771.

**Electronic supplementary material:**

The online version of this article (doi:10.1007/s00535-017-1326-1) contains supplementary material, which is available to authorized users.

## Introduction

Ulcerative colitis (UC), a chronic inflammatory bowel disease (IBD) confined to the colon, is characterized by diarrhea, rectal bleeding, rectal urgency and tenesmus [1]. The prevalence of UC is high in western countries; in USA, the prevalence ranges from 37 to 246 per 100,000 persons [2, 3]. In Japan, the age-standardized prevalence of UC in 2005 was 63.6 per 100,000 persons and it is rapidly increasing with time, although at a comparatively lower rate than the western countries. Growing incidence and emergence of UC in formerly low-risk populations like Japan highlight the effects of the environment and the growing western influence on dietary habits [4, 5]. In addition to westernization, familial traits, genetics and smoking habits also contribute to the development and progression of UC [5].

The conventional therapies used for the management of moderately to severely active UC include 5-aminosalicylate (5-ASA) compounds, corticosteroids, immunomodulators such as 6-mercaptopurine (6-MP), azathioprine (AZA) and cyclosporine. However, many patients fail to achieve adequate response and tolerate conventional therapies [6]. As tumor necrosis factor (TNF)-α, a proinflammatory mediator, plays an integral role in the pathogenesis of UC, treatment with TNF-α antagonist such as infliximab has proven to be effective in moderate to severe UC [7, 8].

Golimumab, a fully human monoclonal anti-TNFα antibody, was approved in the USA and the European Union in 2013 for induction and maintenance treatment of moderately to severely active UC. The approval was based on two phase 3 clinical studies (Program of Ulcerative Colitis Research Studies Utilizing an Investigational Treatment [PURSUIT]-SC, a 6-week induction study [9] and the PURSUIT-Maintenance [PURSUIT-M], a 54-week maintenance study [10]), which evaluated the efficacy and safety of golimumab subcutaneous injection (SC) in moderately to severely active UC patients who were refractory or intolerant to conventional UC therapies. Both the PURSUIT-SC and PURSUIT-M studies also enrolled Japanese patients; however, the small sample size of Japanese patients limits the generalizability of the results in these patients. Hence, the present study was conducted to evaluate the efficacy and safety of golimumab via SC as maintenance therapy in Japanese patients with moderately to severely active UC and to confirm the similarities of golimumab efficacy and safety between this study and those observed in the PURSUIT-M study.

## Methods

### Patients

Japanese patients (both men and women aged ≥18 years) with moderately to severely active UC, as defined by a Mayo score of 6–12 with a local endoscopic subscore ≥2 at baseline of induction phase were eligible if they had an inadequate response to or had failed to tolerate one or more conventional therapies [oral 5-aminosalicylates, oral corticosteroids, azathioprine (AZAs) and/or mercaptopurine] or had demonstrated corticosteroid dependence. All patients in this study were TNF α therapy-naïve. In addition, eligible patients were to have a stool culture negative for enteric pathogens and patients aged ≥45 years were to have undergone a colonoscopy to assess the presence of adenomatous polyps (within 5 years prior to first administration of study agent) and adenomatous polyps were to be removed prior to the first administration of any study agent. Patients with no history of latent or active tuberculosis (TB) prior to screening were included. In the screening phase, infections were assessed by an investigator. Two months prior to the first administration of golimumab, patients were screened for TB using the interferon-gamma release assays (IGRAs): QuantiFERON-TB Gold Testing and T-SPOT.TB test, chest radiographic and lung computed tomography (CT). Patients were also screened for hepatitis B virus (HBV) infections using the HBsAg (HBV surface antigen), anti-HBs (HBV surface antibody) and anti-HBc total (HBV core antibody total) assays.

Patients with a history of severe and extensive colitis who required colectomy; or who had colits limited to 20 cm of the colon or rectum, symptomatic colonic or small bowel obstruction, colonic mucosal dysplasia, extensive colonic resection or any other intra-abdominal surgery within a specified interval prior to screening; or patients having presence of fistula or adenomatous colonic polyps (not removed) were not included in this study.

The institutional review board at the study site approved the protocol and the study was conducted in accordance with the ethical principles that have their origin in the Declaration of Helsinki, consistent with good clinical practices and applicable regulatory requirements. All enrolled patients provided written consent for their participation in the study.

### Study design

**Fig. 1:**
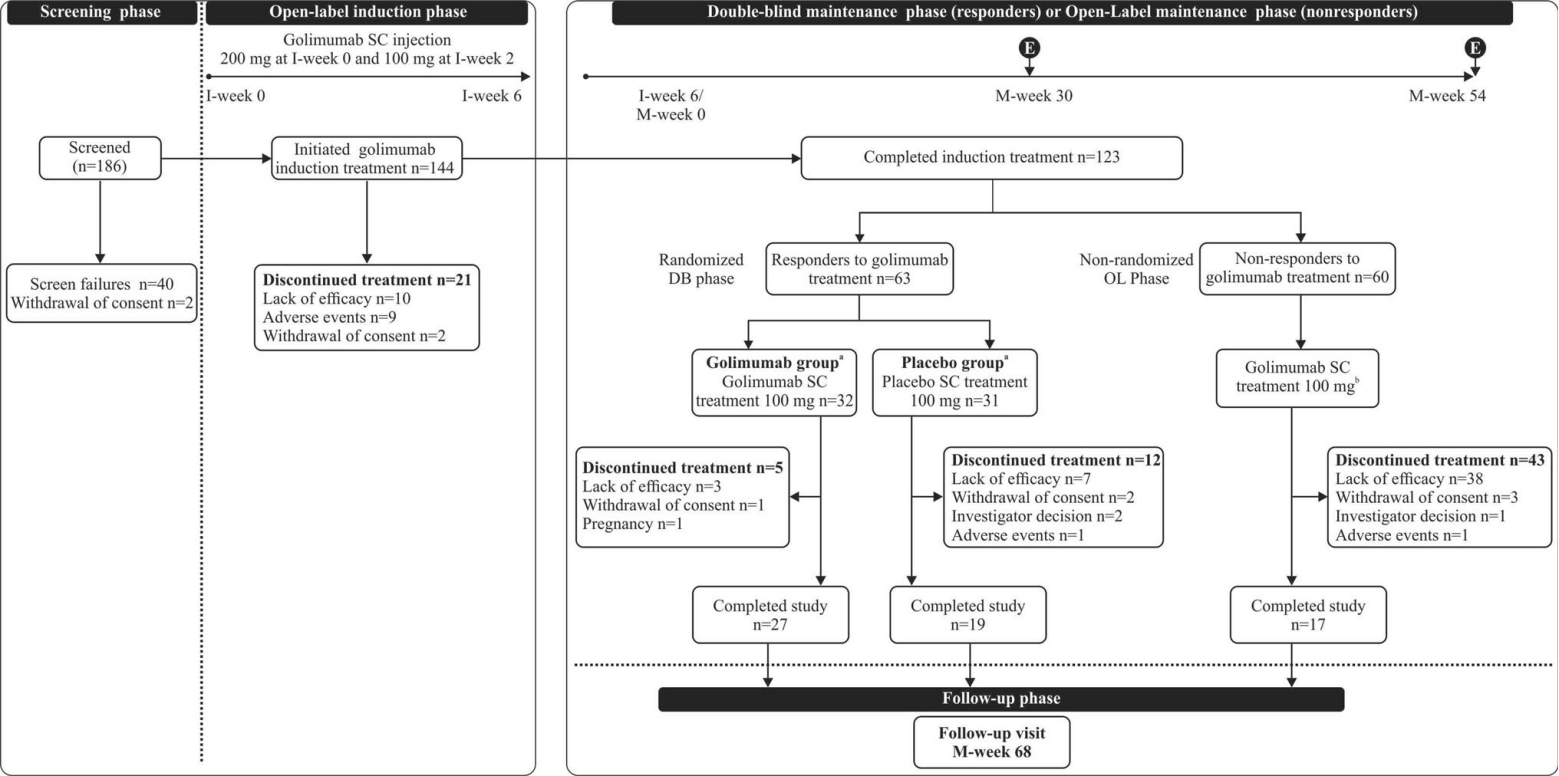
Study design and patient disposition. *E* Primary efficacy evaluation, *DB* double-blind, *I*-*week* induction week, *M*-*week* maintenance week, *OL* open-label, *SC* subcutaneous; ^a^ every 4 weeks through M-week 52; ^b^ patients who responded to golimumab 100 mg at M-week 8 continued to receive golimumab 100 mg every 4 weeks through M-Week 52 at the same dose

This was a phase 3, double-blind, placebo-controlled, 2-arm parallel group, randomized withdrawal study conducted at 49 centers in Japan between February 2013 to January 2016. The study consisted of a 6-week open-label induction phase (I) and a 54-week double blind (DB)-maintenance phase (M; Fig. 1). In the open-label induction period (I-week 0 to I-week 6), all patients received 200 mg of golimumab via SC at I-week 0 and 100 mg at I-week 2. At I-week 6 (i.e. M-week 0), patients who achieved clinical response (CR) entered the DB-maintenance phase as the primary population and were randomized (1:1) to receive either 100 mg of golimumab or an equivalent placebo every 4 weeks through M-week 52. Treatment allocation was based on a computer-generated randomization schedule that used permuted block randomization with stratification factors as clinical remission and corticosteroid use at M-week 0 (yes or no). For patients with a confirmed loss of CR, the following dose adjustments were permitted once during the study: patients on placebo received 100 mg of golimumab every 4 weeks and patients on golimumab treatment continued receiving the same dose every 4 weeks. The study was registered at ClinicalTrials.gov NCT01863771.

Nonresponders to golimumab induction treatment were included in the open-arm [open-label (OL)-maintenance phase] to receive 100 of golimumab via SC at M-week 0 and M-week 4. However, at M-week, eight patients who did not show improvements in Mayo score from I-week 0 were discontinued from the study. All patients had a follow-up at 16 weeks after the last golimumab administration for safety assessments.

Patients on corticosteroid therapy at I-week 0 were continued on the therapy through the induction phase. For patients in CR to golimumab in the induction phase, dose tapering for corticosteroids should have been performed from M-week 0. The recommended rate of corticosteroid tapering was not more than 5 mg/week for patients on a corticosteroid dose >20 mg/day and not more than 2.5 mg/week for patients on a corticosteroid dose ≤20 mg/day.

### Study evaluations and endpoints

#### Primary efficacy endpoint

The primary efficacy endpoint was defined as maintenance of CR through the end of the DB-maintenance phase (M-week 54) in golimumab responders (induction responders). It was assessed using the Mayo score, a composite endoscopic clinical score calculated as a sum of four subscores: stool frequency, rectal bleeding, endoscopy findings and physician’s global assessment. Mayo overall score values range from 0 to 12 with higher scores indicative of severe disease condition [11].

CR was measured as a decrease in the Mayo score by ≥30% and ≥3 points from I-week 0, along with a fall in the rectal bleeding subscore of ≥1 or a rectal bleeding subscore of 0 or 1. Mayo scores were calculated at I-week 0, M-week 0, M-week 30 and M-week 54. In addition, patients experiencing an increase in disease activity (i.e., clinical flare defined as an increase in the partial Mayo score of at least two points from baseline [M-week 0] with an absolute partial Mayo score ≥4 or an absolute partial Mayo score ≥7) at any time during the study were also assessed for loss of CR using Mayo scores. Partial Mayo scores defined as Mayo scores without endoscopic assessments were calculated at all study time points.

#### Secondary endpoints

Clinical remission (defined as a Mayo score of ≤2 points, with no individual subscore >1) and mucosal healing (measured using a Mayo endoscopic subscore of 0 or 1) at both M-week 30 and M-week 54. Other efficacy endpoints were: proportion of patients who maintained clinical remission at both M-week 30 and M-week 54 among patients induced into clinical remission with SC golimumab; proportion of patients achieving clinical remission and eliminating corticosteroid use at M-week 54 among patients receiving concomitant corticosteroids at M-week 0; change in Mayo scores and partial Mayo scores from baselines (I-week 0 and M-week 0); proportion of patients who reported mucosal healing at various time points and change from baseline in corticosteroid use at M-week 30 and M-week 54. Health-related quality of life was assessed using a patient-reported IBD questionnaire (IBDQ), a 32-item self-reported questionnaire that evaluated bowel symptoms, systemic symptoms, social function and emotional functions [12]. Improvements in the IBDQ was assessed in patients who had >20-point improvement in the IBDQ score [13] at M-week 0 from I-week 0. The proportion of patients with sustained improvement in their IBDQ score of >20 points at M-week 30 and M-week 54 was also assessed.

#### Immunogenicity and biomarkers

Immunogenicity was measured by antibody detection and characterization from I-week 0 through the follow-up visit at week 68. Blood samples were collected prior to study drug administration on I-week 0, I-week 6, M-week 28, M-week 30, M-week 52, M-week 54 and M-week 68 (for a final safety evaluation). Assessments for biomarkers such as C-reactive protein (CRP), fecal lactoferrin and calprotectin were also performed.

#### Safety evaluations

Safety and tolerability were assessed through the induction and maintenance phases and involved monitoring treatment-emergent adverse events (TEAEs), laboratory tests (hematology, serum chemistry), ECG, and measurement of vital signs, physical evaluations, injection site evaluations, TB evaluation and infections.

### Statistical analysis

#### Sample size

The number of patients enrolled in this study was dependent on the target population who were randomized into the DB-maintenance phase. Based on the results of the global PURSUIT-M study [10] (with the Japanese subpopulation), the assumed CR would be 30% during the induction phase. Therefore, to ensure a total patient population of 60 during the DB-maintenance phase, a total of 200 patients were required to be enrolled assuming a CR of 30% in the induction phase.

#### Primary efficacy analysis

The primary hypothesis of this study was based on achievement of difference in efficacy between golimumab maintenance treatment (100 mg SC, every 4 weeks, for 52 weeks) and placebo groups as per predefined criterion: CR_100_–CR_P_ ≥8.5%, where CR_100_ and CR_P_ were the point estimates of the proportion of patients in CR through M-week 54 (i.e. M-week 0, 30, 54) in the golimumab and placebo groups. A lower limit of 95% confidence interval (CI) was used to establish the efficacy criterion for the primary endpoint in the current study population consisting of Japanese adult patients, which was established based on the data from CR through M-week 54 between the 100 mg of golimumab group and the placebo group in the global PURSUIT–M study [10].

### Analysis sets

*Efficacy* All patients who received the induction treatment were included in the efficacy full analysis set for the induction phase (FAS-I). The primary analysis set for the DB-maintenance phase (FAS-DB) included the target population, i.e., all patients who were randomized at M-week 0, whereas nonrandomized patients who entered the OL-maintenance phase were included in the open-label maintenance phase full analysis set (FAS-OL). All treatment group comparisons were performed at a two-sided α level of 0.05 and the CI was also estimated. 

The proportion of patients who maintained CR, clinical remission at M-week 30 and M-week 54 with mucosal healing at M-week 30 and M-week 54 and achieved corticosteroid-free clinical remission at week 54 in the FAS-DB were calculated per treatment group. Actual values and change from baseline in the Mayo and partial Mayo scores over time in patients in the FAS-I, FAS-DB and FAS-OL analysis sets were summarized by treatment group.

*Immunogenicity and biomarker assessments* were based on the immune response (IR) analysis set and included patients who received at least one administration of golimumab and had at least one observed IR value after administration. Changes from baseline values (I-week 0 and M-week 0) in CRP concentrations, fecal lactoferrin and calprotectin concentrations (log-transformed) over time were compared between golimumab and placebo at I-week 6 and M-week 16, 30 and 54.

Patient-reported outcomes: patients with >20-point improvement in the IBDQ score at M-week 0 from induction baseline and the proportion of patients with sustained improvement in the IBDQ score from baseline (I-week 0) of >20 points at M-week 30 and M-week 54 were summarized by treatment group.

*Safety* All patients who received at least one administration of induction treatment were included in the safety analysis for the induction phase (SF-I). In the DB-maintenance phase, the target population was included in the DB-maintenance phase safety set (SF-DB), while nonrandomized patients who entered the OL-maintenance phase were included in the OL-safety set (SF-OL). Safety evaluations were summarized descriptively.

## Results

### Demographic and baseline characteristics

A total of 144/186 screened patients (77.4%) initiated golimumab treatment in the induction phase. Of the 144 patients, 123 patients (85.4%) completed the induction phase at I-week 6 and entered the maintenance phases. A total of 63 (43.8%) patients achieved CR to golimumab treatment in the induction phase and were randomized to receive 100 mg of golimumab (32 patients) or to be placed in the placebo group (31 patients) in the DB-maintenance phase as the primary population (Table 1). Furthermore, 60/123 patients who completed the induction phase, but were nonresponders to induction dosing at I-week 6/M-week 0, entered the OL-maintenance phase without randomization. The proportion of patients who discontinued study/terminated the study participation in the maintenance phase was greater in the nonrandomized patients [71.7% (43/60)] than the randomized patients [27.0% (17/63)]. Of the 63 randomized patients, 34 patients were considered treatment failures (golimumab = 10, placebo = 24) and 27 patients (golimumab = 8, placebo = 19) underwent dose adjustments. All 19 patients on placebo earlier underwent dose adjustments to receive 100 mg of golimumab.

**Table 1:** Demographic and baseline characteristics at I-week 0 (full analysis set for the induction, DB-maintenance and OL-maintenance phases)

	Induction phase	DB-Maintenance phase		OL-Maintenance phase
	Golimumab SC 200 mg	Golimumab SC 100 mg	Placebo* 100 mg	Golimumab SC 100 mg
*N*	144	32	31	60
Sex, male, *n* (%)	98 (68%)	19 (59%)	19 (61%)	42 (70%)
Age (years)	42.40 (14.74)	39.30 (12.00)	42.90 (14.41)	42.10 (16.16)
Weight (kg)	61.51 (11.18)	64.59 (14.73)	59.48 (9.73)	60.97 (9.74)
Height (cm)	165.63 (7.50)	163.77 (7.05)	163.49 (6.21)	167.09 (7.77)
BMI (kg/m^2^)	22.38 (3.69)	24.07 (5.44)	22.22 (3.24)	21.78 (2.77)
Disease duration^a^, years	5.08 (0.1;27.4)	5.35 (0.5;24.7)	5.74 (0.3;21.6)	4.57 (0.3;27.4)
Extent of disease, *n* (%)				
Limited to left side of colon	89 (62%)	20 (63%)	19 (61%)	37 (62%)
Extensive	55 (38%)	12 (38%)	12 (39%)	23 (38%)
Mayo score^a^ (0–12)	8.0 (6;12)	8.0 (6;11)	8.0 (6;12)	8.0 (6;11)
Severity of UC disease, *n* (%)				
Moderate	141 (98%)	31 (97%)	30 (97%)	59 (98%)
CRP (mg/L)	4.90 (10.95)	5.31 (14.79)	4.06 (7.74)	4.68 (11.06)
Any UC medication, *n* (%)	141 (98%)	32 (100%)	30 (97%)	59 (98%)
Corticosteroid^#^	42 (29%)	9 (28%)	9 (29%)	19 (32%)
≥20 mg/day P.Eq	12 (8%)	4 (13%)	5 (16%)	3 (5%)
<20 mg/day P.Eq	30 (21%)	5 (16%)	4 (13%)	16 (27%)
Budesonide	0	0	0	0
Immunomodulatory drugs				
6-MP/AZA	64 (44%)	16 (50%)	13 (42%)	28 (47%)
Methotrexate	0	0	0	0
Aminosalicylates	128 (89%)	29 (91%)	27 (87%)	55 (92%)

All values expressed as mean (SD) unless otherwise mentioned

*6-MP* 6-mercaptopurine, *AZA* azathioprine, *BMI* body mass index, *CRP* C-reactive protein, *DB* double-blind, *OL* open-label, *SC* subcutaneous injection, *SD* standard deviation, *UC* ulcerative colitis

^a^Values expressed in median (range)

* Patients who were in clinical response to golimumab induction therapy and were randomized to the placebo in the maintenance phase; #excluding budesonide

For patients enrolled in the induction phase (*n* = 144), the median duration of UC was 5.08 years, the median Mayo score was 8.0 and extensive disease was observed in 38.2% of patients. Concomitant medications at baseline were 5-aminosalicylates (89%), corticosteroids (29%) and immunosuppressive AZA/6-mercaptopurine (46%). No patients were on budesonide therapy or methotrexate therapy concomitantly. Baseline demographics were generally similar among the treatment groups (Table 1).

### Efficacy

#### Induction phase

Of the 144 patients treated with golimumab, 63 patients (43.8%) achieved CR, 27 patients (18.8%) achieved clinical remission and 53 patients (36.8%) achieved mucosal healing at I-week 6. A reduction in the Mayo score was observed from baseline at the end of the induction phase (I-week 6), with a median Mayo score of 6.5 (0 to 11) and a median reduction of −2.0 (−9 to 3). Consistently, a reduction in the partial Mayo score was also observed from baseline with a median partial Mayo score of 4.0 (0 to 9) and median reduction of −1.0 (−8 to 3).

### Maintenance phases

#### Primary endpoint

**Fig. 2:**
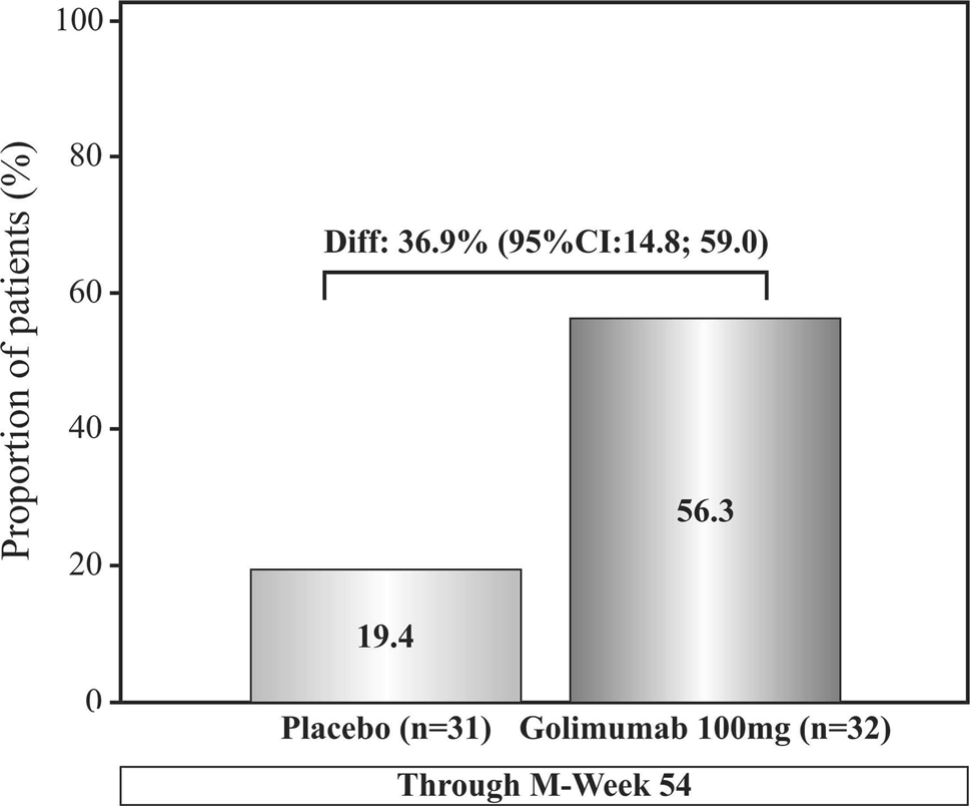
Proportion of patients with clinical response through M-week 54, Full analysis set-DB. *CI* confidence interval, *DB* double-blind, *M*-*week* maintenance week

More patients on golimumab treatment [56.3% (18/32)] maintained CR through M-week 54 than in the placebo group [19.4% (6/31)] and the difference in the proportion of patients between the two treatment groups was 36.9%, (95% CI 14.8; 59.0) which was above the prespecified criterion of ≥8.5% (Fig. 2).

### Other end-points in maintenance phases

**Fig. 3:**
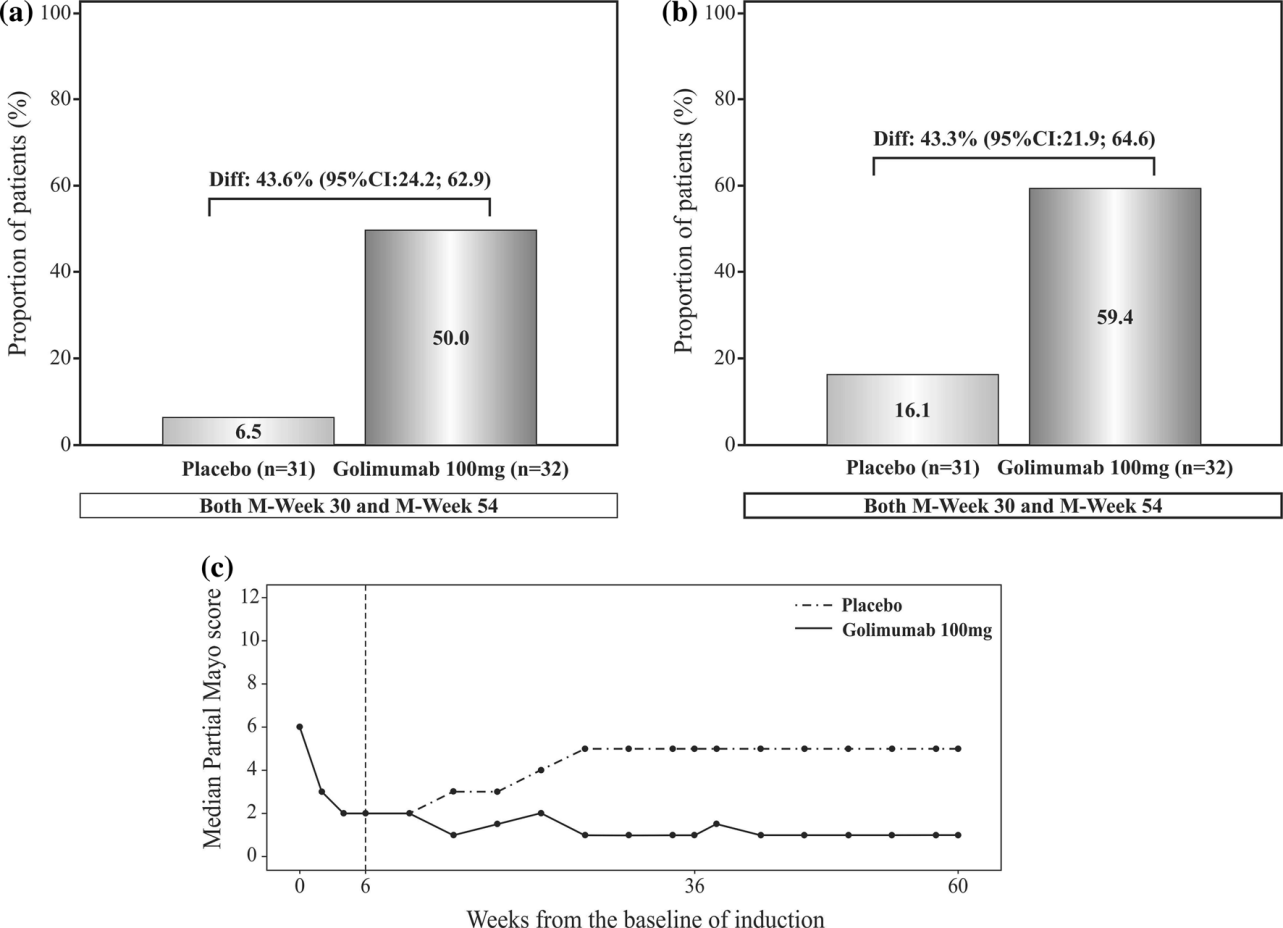
**a** Proportion of patients with clinical remission at both M-week 30 and M-week 54; full analysis set for the DB-maintenance phase. **b** Proportion of patients with mucosal healing at both M-week 30 and M-week 54; full analysis set for the DB-maintenance phase. **c** Change of partial Mayo score overtime; full analysis set for the DB-maintenance phase. *CI* confidence interval, *DB* double-blind, *I*-*week* induction week, *M*-*week* maintenance week

A higher proportion of responders in the golimumab group [50.0% (16/32)] achieved clinical remission at both M-week 30 and M-week 54 as compared with patients in the placebo group [6.5% (2/31)] and the difference in the proportion of patients between the two treatment groups was 43.6% [95% CI: 24.2; 62.9, (Fig. 3a)]. Of the 63 patients who were randomized in the DB-maintenance phase, 14/32 golimumab patients and 13/31 placebo patients were in clinical remission at the baseline visit of the DB-maintenance phase M-week 0. Among these patients, 9/14 (64.3%) golimumab patients and 2/13 (15.4%) placebo patients were in clinical remission at both M-week 30 and M-week 54. More patients on golimumab treatment [59.4% (19/32)] experienced mucosal healing than patients on placebo [16.1% (5/31)] at both M-week 30 and M-week 54 and difference in the proportion of patients between the two treatment groups was 43.3% [95% CI 21.9; 64.6 (Fig. 3b)].

The Mayo scores remained stable at M-week 30 and M-week 54 for patients on golimumab treatment but a median increase of 4.0 (−2 to 8) and 5.0 (−3 to 8) at M-week 30 and M-week 54, respectively, was observed for patients receiving placebo treatment. The partial Mayo score decreased from 6 at I-Week 0 to 2 at I-week 6/M-week 0 in the induction phase; a further decrease to 1 at M-week 8 was observed and was maintained at 1 almost through M-week 54 in the golimumab treatment group (Fig. 3c). Consistent with the Mayo score, the partial Mayo score also remained stable between baseline and M-week 30 and M-week 54 in the golimumab 100 mg group and a median increase of 3.0 (−2 to 6) was observed at both M-week 30 and M-week 54 from baseline in the placebo group (Table 2).

**Table 2:** Mayo score, partial Mayo score, mucosal healing and corticosteroid use at M-week 30 and M-week 54 (full analysis set for the DB-maintenance phase and OL-maintenance phase)

	DB-Maintenance phase^#^		OL-Maintenance phase^#^
	Golimumab SC 100 mg	Placebo 100 mg	Golimumab SC 100 mg
*N*	32	31	60
Mayo score at baseline	3.0 (0;8)	3.0 (0;7)	8.0 (4;11)
Change in the Mayo Score from baseline			
M-week 30^a^	0.0 (−5;6)	4.0 (−2;8)	0.0 (−8;2)
M-week 54^a^	−0.5 (−6;6)	5.0 (−3;8)	0.0 (−10;6)
Mucosal healing, *n* (%)			
M-week 30^a^	19 (59%)	8 (26%)	9 (15%)
M-week 54^a^	20 (63%)	5 (16%)	9 (15%)
Corticosteroid use at baseline	15.0 (1.0;20.0)	15.0 (4.6;30.0)	7.50 (2.5;20.0)
Change in corticosteroid use			
M-week 30^a^	-2.50 (-20.0;0.0)	0.0 (-30.0;0.0)	0.0 (-10.0;0.0)
M-week 54^a^	-1.0 (-20.0; 0.0)	0.0 (-30.0;0.0)	0.0 (-12.0;0.0)
C-reactive protein concentration at baseline (mg/L)	0.36 (0.1;24.0)	0.26 (0.1;22.8)	1.16 (0.1;29.8)
Change in C-reactive protein concentration (mg/L)			
M-week 30^a^	0.08 (−9.7;11.2)	0.25 (−9.7;13.4)	−0.065 (−22.8;77.0)
M-week 54^a^	0.035 (−9.7; 11.2)	0.250 (−9.7;17.2)	−0.02 (−22.8;77.0)
Fecal lactoferrin concentration at baseline (µg/g)^b^	1.20 (−0.9;2.7)	0.78 (−0.5;3.0)	1.89 (−0.5;3.4)
Change in fecal lactoferrin concentration (µg/g)^b^			
M-week 30^a^	0.00 (−3.2;3.0)	0.79 (−2.0;3.1)	0.05 (−3.1;1.3)
M-week 54^a^	−0.04 (−2.9;3.0)	0.76 (−2.7;3.1)	−0.01 (−4.8;1.3)
Fecal calprotectin concentration at baseline (mg/kg)^b^	1.99 (1.2;2.9)	1.88 (1.2;3.0)	2.52 (1.2;3.0)
Change in fecal calprotectin concentration (mg/kg)^b^			
M-week 30^a^	0.0 (−1.6;1.3)	0.48 (−1.2;1.7)	0.05 (−1.6;1.1)
M-week 54^a^	0.0 (−1.6;1.3)	0.54 (−1.7;1.7)	0.06 (−1.8;1.1)

All values expressed as median (range) unless otherwise mentioned

*FAS-DB* double-blind maintenance phase full analysis set

^a^Patients who had a missing value for a parameter at any time point had their last available value carried forward to that time point

^b^Log-transformed values

^#^Baseline is defined as maintenance M-week 0, *DB* double-blind, *OL* open-label, *SC* subcutaneous injection, *SD* standard deviation

In the OL-maintenance phase, the median Mayo scores remained stable at M-week 30 [7.0 (0 to 10)] and M-week 54 [7.0 (0 to 11)] in the nonrandomized patients. Minimal decrease in the median mayo score from baseline was observed at M-week 8 with a median decrease of −1.0 (−6 to 3). Consistent with the Mayo score, change in partial Mayo scores for the nonrandomized patients was also minimal. A median decrease in partial Mayo scores [−1.0 (–4 to 3)] was observed at M-week 8 which remained stable through M-week 54. Of the 60 nonrandomized patients, 13 (21.7%) patients achieved mucosal healing at M-Week 8 and 9/60 (15.0%) patients achieved mucosal healing at both M-week 30 and M-week 54.

### Corticosteroid use during the DB-maintenance phase

Of the 63 randomized patients, 18 patients (each treatment arm = 9), received corticosteroid therapy at baseline M-week 0. Although the median average daily corticosteroid dose was similar in the two treatment groups, at M-week 54, the number of patients in clinical remission and not receiving concomitant corticosteroids was higher in the golimumab treatment [55.6% (5/9)] as compared with placebo treatment [11.1% (1/9)]. A greater reduction from baseline in the mean daily corticosteroid dose was observed in the golimumab group (M-week 30 = reduction of 7.19 mg/kg P.Eq; M-week 54 = 6.50 mg/kg P.Eq) than in the placebo group (M-week 30 = reduction of 6.67 mg/kg P.Eq; M-week 54 = 3.33 mg/kg P.Eq).

### Biomarkers and immunogenicity

The median CRP concentration at I-week 0 was 1.585 mg/L for all patients in the induction phase. At I-week 6, the median CRP concentration decreased by 0.190 mg/L from baseline. At M-week 0, the median CRP concentrations were comparable across the treatment groups and had decreased from I-week 0 in both groups; however, the median CRP concentrations in the golimumab 100 mg group were lower than that in the placebo group at M-week 16 (0.075 mg/L vs. 0.240 mg/L), M-week 30 (0.080 vs. 0.250 mg/L) and M-week 54 (0.035 vs. 0.250 mg/L).

The median fecal lactoferrin concentration at I-week 0 was 1.88 for all patients in the induction phase. At I-week 6, the median fecal lactoferrin concentration decreased by 0.30 from baseline. The median fecal lactoferrin concentrations at M-week 0 were higher in the golimumab 100 mg group (1.20) than in the placebo group (0.78). From M-week 0, the change in median fecal lactoferrin concentrations remained stable in both groups: golimumab 100 mg group vs. placebo group at M-week 30 (0 vs. 0.79) and M-week 54 (−0.04 vs. 0.76).

The median calprotectin concentration at I-week 0 was 2.70 for all patients in the induction phase. At I-week 6, the median fecal calprotectin concentration decreased by 0.19 from baseline. At M-week 30 and M-week 54, the change from baseline in the median fecal calprotectin concentrations remained stable from M-week 0 in the golimumab 100 mg group whereas the concentrations increased in the placebo group (Table 2).

Of all the 144 golimumab-treated patients in the induction phase, no patient was positive for antibodies. During the DB-maintenance phase, 4/63 were positive for antibodies to golimumab (4/31 patients from placebo group). Among the nonrandomized patients receiving golimumab treatment in the OL-maintenance phase, 1/60 patient was positive for antibodies. All five samples showed neutralizing activity against golimumab.

### Patient-reported outcomes

At the end of the induction phase, the median decrease in the IBDQ scores at I-week 6 from I-week 0 was 11.0. A higher proportion of patients on golimumab treatment (55.0%, 11/20 patients) had >20-point improvement in the IBDQ score at M-week 0 from I-week 0 as compared to placebo (22.2%, 6/27) and was maintained through M-Week 54. During the DB-maintenance phase, at M-week 30 and M-week 54, the median change in all IBDQ dimension scores from M-week 0 was lower in the golimumab 100 mg group than in the placebo group, suggesting a maintained improvement in their IBDQ dimension scores achieved during induction. In the OL-maintenance phase, 10 patients had >20-point improvement in the IBDQ score at M-week 0 from I-week 0. Of these, 20% (2/10) of patients maintained >20-point improvement in the IBDQ score through M-week 54.

### Safety and tolerability

#### Induction phase

Of the 144 patients enrolled in the induction phase, 65 patients (45.1%) reported ≥1 TEAE, with nasopharyngitis (17/144; 11.8%) and worsening of UC (9/144; 6.3%) being the most frequent. A total of 5 patients (3.5%) reported serious TEAEs [UC = (4/144; 2.5%) and cytomegalovirus colitis = (1/144; 0.7%)]. A total of 10 patients discontinued the study due to TEAEs, with the most common TEAE being worsening of UC (8/144; 5.6%). Decreased absolute lymphocyte count was observed in 12/144 (8.3%) patients and was the most common markedly abnormal hematology observation.

#### Maintenance phase (DB and OL)

During the DB-maintenance phase, the proportion of patients experiencing ≥1 TEAE in the golimumab 100 mg group was 97% (31/32) and in the placebo group, it was 71.0% (22/31). The most frequently (>10%) reported TEAEs in the golimumab 100 mg group were nasopharyngitis (53.1%) and injection site erythema (15.6%). Serious TEAEs were reported in 3.1% (1/32, Takayasu’s arteritis) of patients in the golimumab 100 mg group as compared with 12.9% (4/31, colitis ulcerative, hand fracture, joint dislocation and cerebral infarction) of patients in the placebo group. Among the randomized patients, 35.5% (11/31) patients on placebo and 65.6% (21/32) patients treated with golimumab reported at least one treatment-emergent infection, with nasopharyngitis as the most frequently reported infection. Injection site reactions were reported only by patients receiving golimumab 18.8% (6/32) with injection site erythema [15.6% (5/32)] being the most frequent. Reduction in lymphocytes was reported in 12/30 (40%) patients in the placebo group and 4/32 (12.5%) of patients in the golimumab 100 mg group.

During the OL-maintenance phase, 78.3% (47/60) of the nonrandomized patients experienced TEAEs. Serious TEAEs were reported in 6/60 patients; worsening of UC was observed in 5 patients and infection was observed in 5 patients (Table 3). Of the 60 nonrandomized patients, 26 (43.3%) experienced at least one infection, with nasopharyngitis 21.7% (13/60) being the most frequently reported infection. Injection site reactions were reported in only 3.3% (2/60) of patients. Decreased lymphocytes were reported in 14/58 (24.1%) non-randomized patients and was the most common markedly abnormal postbaseline hematology observation.

**Table 3:** Summary of treatment-emergent adverse events (full analysis set for the DB-maintenance phase and OL-maintenance phase)

	DB-Maintenance phase		OL-Maintenance phase
	Golimumab SC 100 mg	Placebo 100 mg	Golimumab SC 100 mg
*N*	32	31	60
Average duration of follow-up (weeks)	49.0	24.2	21.5
Average exposure (number of administrations)	12.6	6.4	5.5
Number of patients reporting ≥1 incidence			
Any TEAE	31 (96.9%)	22 (71.0%)	47 (78.3%)
Serious TEAE	1 (3.1%)	4 (12.9%)	6 (10.0%)
Reasonably related TEAEs	11 (34.4%)	2 (6.5%)	8 (13.3%)
Infections	21 (65.6%)	11 (35.5%)	26 (43.3%)
Injection site reactions	6 (18.8%)	0	2 (3%)
Erythema	5 (15.6%)	0	1 (1.7%)
Gastrointestinal disorders	5 (15.6%)	5 (16.1%)	18 (30.0%)
Colitis ulcerative	0	2 (6.5%)	6 (10.0%)
Infections and infestations	20 (62.5%)	13 (41.9%)	3 (5%)
Nasopharyngitis	17 (53.1%)	7 (22.6%)	14 (23.3%)
Influenza	4 (12.5%)	1 (3.2%)	3 (5.0%)
Conjunctivitis	1 (3.2%)	1 (3.2%)	1 (1.7%)
Oral herpes	1 (3.1%)	3 (9.7%)	2 (3.3%)

All values expressed as n (%) unless otherwise mentioned

*DB* double blind, *OL* open-label, *SC* subcutaneous injection, *SD* standard deviation, *TEAE* treatment-emergent adverse event

Overall, no incidences of active TB or malignancy and no deaths were reported during this study. No clinically relevant changes from baseline in the hematology parameters and clinical laboratory parameters were observed during the study..

## Discussion

Subcutaneous injection of golimumab, a fully human anti-TNFα antibody, has demonstrated clinical efficacy in recent double-blind, randomized placebo-controlled global studies by effectively achieving and maintaining CR and remission in patients with moderate to severely active UC [9, 10]. However, in these global studies, no definitive conclusions about the effectiveness of golimumab SC as a maintenance therapy in Japanese patients could be drawn due to the limited number of patients from this region. The current study was the first to assess the efficacy and safety of golimumab SC as both induction and maintenance therapy particularly in Japanese patients. In the current study, efficacy and safety of golimumab therapy was assessed in UC patients who were refractory or intolerant to 5-ASAs, corticosteroids and/or immunomodulators. Consistent with the results of the global PURSUIT-M study, CR was achieved with golimumab therapy in 56.3% of induction responders through M-week 54 during the DB-maintenance phase. Furthermore, golimumab treatment showed 36.9% more efficacy compared with the placebo; thus, the primary efficacy endpoint was achieved based on the predetermined criterion for efficacy (CR_100_–CR_P_ ≥8.5%). The safety results indicated that, consistent with the global studies, golimumab was well-tolerated in this patient population.

At the end of the induction phase, 43.8% of UC patients achieved CR to golimumab. These results are generally consistent with a global induction study wherein a similar proportion of patients (51.0%) achieved CR with golimumab SC 200 mg at the end of a 6-week induction phase [9]. The Japanese subpopulation of the PURSUIT-SC study, however, showed a lower CR of 26.1% during the induction phase as compared with the CR observed in the present study (43.8%) [14]. During the DB-maintenance phase, the observation of CR with golimumab 100 mg in 56.3% of patients in the maintenance phase of this study is also comparable with the global golimumab SC maintenance study (49.7%) [10].

Results of secondary endpoints including clinical remission status and mucosal healing at both M-week 30 and M-week 54 further substantiate the efficacy of golimumab therapy in this study. Clinical remission observed during the induction phase was maintained at M-week 30 and M-week 54 in golimumab-treated patients as compared with placebo-treated patients. A decrease in the Mayo score and partial Mayo score obtained at baseline of maintenance phase from I-week 0 were maintained through the end of study. Mucosal healing was achieved by more than 50% patients at M-week 30 and M-week 54 as compared with placebo, further supporting the efficacy of golimumab 100 mg over placebo. Mucosal healing determined by the endoscopy subscore of 0 or 1 [15] has emerged as one of the most significant treatment goals in IBD such as UC and Crohn’s disease, as it assesses disease activity and helps predict sustained clinical remission. Thus, with similar CR, clinical remission and mucosal healing observed in the present study, the current results of golimumab treatment in the Japanese population are comparable with the overall global population.

Currently, infliximab and adalimumab are approved for the biological therapy of UC in Japan. Golimumab is the latest addition to the anti-TNF class of drugs and offers some advantages over the conventional biological treatments. In addition, golimumab treatment may prove to be beneficial over the other treatments such as adalimumab in terms of a longer dosing schedule (4- vs. 2-week) for maintenance treatment. An important point to consider is the relatively convenient ease of administration of golimumab SC, as compared with other treatments like infliximab, which require intravenous administration in hospital settings [16].

One of the key roles of an ideal UC therapy is to reduce the need for concomitant corticosteroid therapy and lower the consequent side effects. Several agents such as azathioprine and 5‐ASA have been evaluated for their ability to discontinue steroid therapy and induce steroid-free remission [17]. In this study, golimumab reduced the mean daily corticosteroid dose at both M-week 30 and M-week 54 from baseline and 55.6% patients on golimumab treatment achieved clinical remission and discontinued corticosteroid use at M-week 54. Patient-related improvements in disease status, quality of life and patient preferences as measured by IBDQ score are gaining impetus and IBDQ score is now being used as a measure of clinical effectiveness in UC [18]. An increase in the median IBDQ score was noted at the end of the induction phase and was maintained through M-week 54. This tendency is consistent with that observed in the PURSUIT-M study [10].

Inflammation is indicated as an underlying cause of UC and various treatment regimens have been evaluated for their effects on suppressing these subclinical inflammations [19]. The inflammatory biomarker CRP and stool biomarkers with enhanced specificity for intestinal inflammation such as fecal lactoferrin, and fecal calprotectin [19] were also assessed to estimate the effects of golimumab in reducing the degree of mucosal inflammation. In the current study, the initial reduction in these biomarker levels was observed at the end of the induction phase and was maintained through M-week 54, suggesting the positive effects of golimumab SC in controlling inflammation associated with UC. The incidence of antibodies to golimumab was low through M-week 54, consistent with that observed in the global study [10].

No new safety signals were observed with golimumab treatment in this UC patient population from Japan. There were no deaths and no cases of active TB or malignancy reported during this study. The pattern of TEAEs, serious TEAEs, infections and injection site reactions during this study were comparable to the global study [9, 10].

The findings of this study are limited by the fact that it was not statistically powered to detect a difference between the golimumab and placebo groups for the end point of maintenance of clinical remission. All between-group comparisons were descriptive and no formal statistical tests were applied to detect the differences between the two treatment groups.

To summarize, the encouraging efficacy and safety outcomes of the current study and the two global studies collectively demonstrate the consistent clinical benefit and positive benefit/risk profile of golimumab treatment. Thus, golimumab can be an additional valuable treatment option for moderate to severe UC in patients from Japan.

## Electronic supplementary material

Below is the link to the electronic supplementary material.

Supplementary material 1 (DOCX 34 kb)
