# Supplementary material for: Efficacy and safety of golimumab 52-week maintenance therapy in Japanese patients with moderate to severely active ulcerative colitis: a phase 3, double-blind, randomized, placebo-controlled study-(PURSUIT-J study)
Source: J Gastroenterol. 2017 Mar 21;52(10):1101–11. doi: 10.1007/s00535-017-1326-1 (PMC5606947; doi:10.1007/s00535-017-1326-1)
Supplement: Supplementary file 1 — Supplementary material 1 (DOCX 34 kb) [file 535_2017_1326_MOESM1_ESM.docx]

Supplementary information

**List of investigators**

A Ando, Shiga University of Medical Science Hospital, Otsu, Shiga; T Ashida, Sapporo Higashi Tokushukai Hospital, Sapporo, Hokkaido; H Hanai, Hamamatsu South Hospital, Minamiku, Hamamatsu; T Ishida, Oita Red Cross Hospital, Oita City, Oita; H Ito, Kinshu-kai Infusion Clinic, Osaka City, Osaka; T Matsumoto, Kyushu University Hospital, Fukuoka city, Fukuoka; S Motoya, Hokkaido P.W.F.A.C. Sapporo-Kosei General Hospital, Sapporo-shi, Hokkaido; S Nakamura, Hyogo College of Medicine, Nishinomiya, Hyogo; Y Sameshima, Sameshima Hospital, Kagoshima-city, Kagoshima; Y Suzuki, Toho University, Sakura Medical Center, Sakura-shi, Chiba; K Watanabe, Osaka City University Hospital, Osaka City, Osaka; H Yamagami, Osaka City University Hospital, Osaka City, Osaka; T Yamamoto, Yokkaichi Social Insurance Hospital, Department of Surgery, Yokkaichi, Mie; K Yao, Fukuoka University Chikushi Hospital, Chikushi, Fukuoka.; N Horiki, Mie University Hospital, Mie Prefecture, Tsu; H Iijima, Osaka University Hospital Department of Medicine, Gastroenterology and Hepatology, Suita; M Iwabuchi, National Hospital Organization Sendai Medical Center, Miyagi Prefecture, Sendai; T Kanai, Keio University Hospital, Shinjuku-ku, Tokyo; R Kunisaki, Yokohama City University Medical Center, Kanagawa, Minami-ku, Yokohama; A Maemoto, Sapporo Higashi Tokushukai Hospital, Sapporo, Hokkaido; K Matsuoka, Keio University Hospital, Shinjuku-ku, Tokyo; S Nakamura, Hyogo College of Medicine 1-1 Mukogawacho, Nishinomiya, Hyogo; T Osada, Juntendo University Hospital, Tokyo; K Sugimoto, Hamamatsu University Hospital, Shizuoka Higashi-ku, Hamamatsu; S Tanaka, Hiroshima University Hospital, Hiroshima, Minami-ku; K Matsuoka, Kei University Hospital, Shinjuku, Tokyo; E Motohiro, Kyushu University Hospital, Fukuoka City, Fukuoka; K Takanori, Keio University Hospital, Shinjuku-ku, Tokyo; T Yamamoto, Yokkaichi Hazu Medical Center, Yokkaichi, Mie; K Yao, Fukuoka University Chikushi Hospital, Chikushi, Fukuoka.
